# Supplementary material for: The Systems Biology Research Tool: evolvable open-source software
Source: BMC Syst Biol. 2008 Jun 29;2:55. doi: 10.1186/1752-0509-2-55 (PMC2446383; doi:10.1186/1752-0509-2-55)
Supplement: Additional file 1 — SBRT Archive. An archive of the current version of the Systems Biology Research Tool. [file 1752-0509-2-55-S1.zip › sbrt-1.4.0/doc/developers_guide/api/sbrt/kernel/fba/comp/ExtremeCurrentComputer.html]

ExtremeCurrentComputer


|  |  |  |
| --- | --- | --- |
| **Overview** \| **Package** \| **Class** \| **Use** \| **Tree** \| **Deprecated** \| **Index** \| **Help** | |  |
| **PREV CLASS**   **NEXT CLASS** | **FRAMES**    **NO FRAMES**     **All Classes** |  |
| SUMMARY: NESTED \| FIELD \| CONSTR \| METHOD | DETAIL: FIELD \| CONSTR \| METHOD |  |


---


## sbrt.kernel.fba.comp Interface ExtremeCurrentComputer

**All Known Implementing Classes:**: SlpExCurrComputerV2

---

``` public interface ExtremeCurrentComputer ```

This interface is used to represent extreme current computers. An extreme
current is very similar to an extreme pathway. For a fluxome containing only
irreversible reactions (including exchange reactions), no extreme pathways
exists, only extreme currents. Their interpretation is essentially
equivalent.

**Author:**
:   This interface was written and documented by Jeremiah Wright while in
    the Wagner lab.

---

| **Method Summary** | |
| --- | --- |
| `SparseDoubleMatrix<?,java.lang.String>` | `getMatrix(IrrevRxnFluxome fluxome)`             Returns the extreme current matrix for the provided fluxome. |
| `SparseDoubleMatrix<?,java.lang.String>` | `getMatrix(IrrevRxnFluxome fluxome, MutableProgressMonitor pm)`             Returns the extreme current matrix for the provided fluxome. |
| `ProgressMonitor` | `getProgress()`             Returns a progress monitor for this extreme current computer. |
| `int` | `getTotal(IrrevRxnFluxome fluxome)`             Returns the total number of operations required to compute the extreme currents of the provided fluxome. |

**Method Detail**

### getMatrix

```
SparseDoubleMatrix<?,java.lang.String> getMatrix(IrrevRxnFluxome fluxome)
```

:   Returns the extreme current matrix for the provided fluxome.

    :   **Parameters:**: `fluxome` - the fluxome for which all extreme currents will be computed. **Returns:**: the extreme current matrix for the provided fluxome.

---


### getProgress

```
ProgressMonitor getProgress()
```

:   Returns a progress monitor for this extreme current computer.

    :   **Returns:**: a progress monitor for this extreme current computer.

---


### getTotal

```
int getTotal(IrrevRxnFluxome fluxome)
```

:   Returns the total number of operations required to compute the extreme
    currents of the provided fluxome. The number returned by this method is
    meant for use with progress monitors.

    :   **Parameters:**: `fluxome` - the fluxome for which the number of operations will be returned. **Returns:**: the total number of operations required to compute the extreme currents of the provided fluxome.

---


### getMatrix

```
SparseDoubleMatrix<?,java.lang.String> getMatrix(IrrevRxnFluxome fluxome,
                                                 MutableProgressMonitor pm)
```

:   Returns the extreme current matrix for the provided fluxome.

    :   **Parameters:**: `fluxome` - the fluxome for which all extreme currents will be computed.: `pm` - the progress monitor to be used during computation of extreme currents. **Returns:**: the extreme current matrix for the provided fluxome.


---


|  |  |  |
| --- | --- | --- |
| **Overview** \| **Package** \| **Class** \| **Use** \| **Tree** \| **Deprecated** \| **Index** \| **Help** | |  |
| **PREV CLASS**   **NEXT CLASS** | **FRAMES**    **NO FRAMES**     **All Classes** |  |
| SUMMARY: NESTED \| FIELD \| CONSTR \| METHOD | DETAIL: FIELD \| CONSTR \| METHOD |  |


---
